# Oral Microorganisms and Biofilms: New Insights to Defeat the Main Etiologic Factor of Oral Diseases

**DOI:** 10.3390/microorganisms10122413

**Published:** 2022-12-06

**Authors:** Martinna Bertolini, Raphael Cavalcante Costa, Valentim Adelino Ricardo Barão, Cristina Cunha Villar, Belen Retamal-Valdes, Magda Feres, João Gabriel Silva Souza

**Affiliations:** 1 Department of Periodontics and Preventive Dentistry, School of Dental Medicine, University of Pittsburgh, Pittsburgh, PA 15106, USA; 2 Department of Prosthodontics and Periodontology, Piracicaba Dental School, University of Campinas (UNICAMP), Piracicaba 13083-970, SP, Brazil; 3 Division of Periodontics, Department of Stomatology, School of Dentistry, University of São Paulo, São Paulo 05508-010, SP, Brazil; 4 Dental Research Division, Guarulhos University, Guarulhos 05508-010, SP, Brazil; 5 Center for Clinical and Translational Research, Forsyth Institute, Boston, MA 02142, USA; 6 Dental Science School (Faculdade de Ciências Odontológicas—FCO), Montes Claros 39401-303, MG, Brazil; 7 Oncovida Cancer Research Center, Montes Claros 39400-111, MG, Brazil

## 1. Introduction

The oral cavity presents a highly diverse community of microorganisms due to the unique environmental conditions for microbial adhesion and growth [1]. The range of habitats and substrates for microbial colonization in the oral cavity provides a diverse biogeography for indigenous micro-organisms to live in a symbiotic state with the host [2]. 

**Figure 1:**
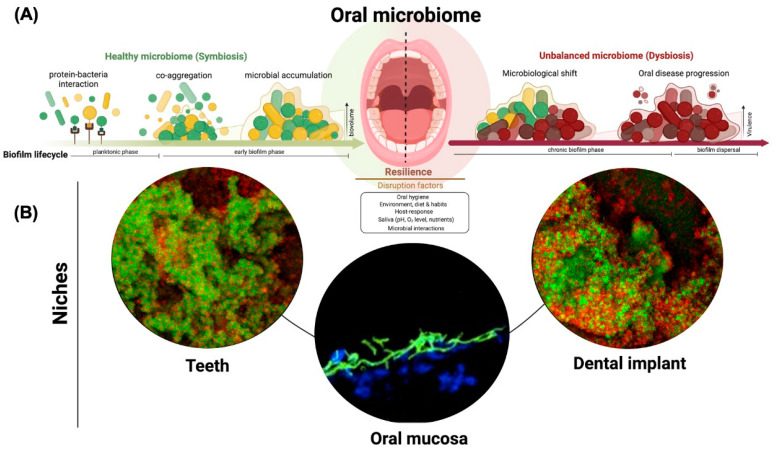
The biofilm formation in the oral cavity. (**A**) Oral surfaces (tooth and mucosal tissues) and any material (i.e., dental implant) inserted in the mouth act as a substrate for microbial adhesion and accumulation. Proteins immediately coat the surfaces from oral fluids (i.e., saliva and plasma), which is the main mediator of microbial adhesion through adhesin-receptor interactions. Then, initial colonizers adhere to the surfaces, binding to the protein layer, followed by co-aggregation processes and interaction between different species to promote biofilm accumulation. Different factors have been identified to disrupt the symbiotic state, leading to the overgrowth of putative pathogens (dysbiosis) able to trigger/foster oral diseases. (**B**) Polymicrobial biofilms can accumulate and induce oral diseases in different habitats of the oral environment, such as teeth, oral mucosa, and dental implants, as shown by microscopic confocal images (bottom) (created using Bio-Render^®^).

Oral microorganisms can colonize biotic [3] and abiotic [4] surfaces, including teeth, soft tissues, dental implants and restorative materials. Different intraoral niches harbor distinct microbial communities, which are also modulated by surface properties and the local micro-environment [5,6,7,8]. The first step of microbial colonization is the salivary pellicle protein adsorption by all available oral surfaces [9,10,11], which is followed by microbial adhesion and growth. As microorganisms accumulate, they form structures known a\s biofilms, which are highly organized microbial communities enmeshed in a three-dimensional extracellular matrix [7,12]. The biofilm structure provides several advantages to colonizing species, such as protection against antimicrobials and host-defense, enhanced co-aggregation, and interaction properties [13,14] (Figure 1A). These protection mechanisms between microorganisms makes biofilms challenging therapeutic targets [15]. 

Oral biofilms are the main etiologic factor of a variety of oral diseases such as dental caries, periodontal diseases, implant-related infections, and oropharyngeal candidiasis [16,17]. Among these diseases, dental caries and periodontal disease are highly prevalent in the world’s population [18], representing an economic burden for healthcare systems worldwide [19]. Oropharyngeal candidiasis commonly affects patients with weakened or immature immune systems [20,21], often leading to systemic bloodstream infection (candidemia) in patients under intensive medical care [22]. Although very distinct, all these conditions have the complex polymicrobial biofilm as a common etiological factor. Changes in endogenous bacterial levels or proportions, in the oral cavity environment and in the host response can transform a healthy-associated into a disease-associated microbial community [5,23,24,25,26] (Figure 1B).

The rapid growth in knowledge about the oral microbiome composition, functionality, and its interactions with the host observed in recent years opens up new avenues for developing effective treatment strategies for oral infections [27]. This special issue calls for papers that focus on the composition of oral biofilms associated with various disease conditions and their effective modulation toward a healthy state. The ultimate goal is to increase knowledge about the host-microbial interplay and the use of traditional or novel therapies for treating oral infections. 

### 1.1. Dental Caries: Biofilm-Sugar-Dependent Disease

Dental caries is a biofilm and sugar-induced disease [28,29]. Recently, data from the 2017–2020 National Health and Nutrition Examination Survey (NHANES) estimates the prevalence of untreated active caries in more than 1 in 5 adults (21.3%) within the US population [30]. The disease is caused by ubiquitous and endogenous oral bacterial species that accumulate on dental surfaces. When certain oral microorganisms are exposed to sugars from the diet, there is a disruption in the physiologic and chemical equilibrium between tooth mineral and ions concentration in the biofilm fluid. These bacterial species metabolize sugar and produce acids that reduce the biofilm fluid pH. The drop in PH leads to a mineral discrepancy between acidic biofilm fluid and tooth, resulting in loss of tooth mineral composition, which can be clinically noted as loss of tooth structure and cavitations [28].

*Streptococcus mutans* has been indicated as the main microorganism related to dental caries development. This microorganism can grow in acidic environments with low pH (aciduric) and has a high capacity for acid production (acidogenic), important factors for caries lesion progression [31]. Moreover, *S. mutans* has three exoenzymes glucosyltransferases (Gtfs) that hydrolyze sucrose from diet and synthesize glucan polymers from the resulting glucose, which contribute to the matrix scaffold and the three-dimensional architecture of biofilms [32]. These extracellular polymers are critical virulence factor to enhance biofilm growth and antimicrobial resistance and induce bio-film-induced diseases [14]. In addition, these polymers mediate the cross-kingdom interaction with Candida albicans, promoting biofilm growth, virulence, and disease progression [33]. Therefore, important factors related to *S. mutans* and extracellular polymers’ role, molecular mechanisms, and the effect of these factors on disease progression, also need to be better elucidated.

When considering treatment modalities for dental caries, toothpastes containing stannous fluoride (SnF_2_) have been around since the 1950s, with proof of its anti-caries effects, as reviewed by others [34]. However, only recently, with the advent of modern imaging techniques and microbiome composition analysis, have researchers been able to fully dissect the biological changes in oral biofilms exposed to SnF_2_, showing that SnF_2_-containing toothpaste changes the biofilm architecture and gene expression, making the biofilm less adhesive and non-virulent [35].

When considering newly developed techniques to prevent dental caries, engineering of the acquired enamel pellicle using salivary peptides could modulate the steps of biofilm formation, leading to anti-caries effect. Recently, the use of engineered salivary peptides has been tested on enamel demineralization against a cariogenic Streptococcus mutans in vitro biofilm, with promising results [36]. In addition, medicinal plant extracts [37] have gained recent interest as some present anti-bacterial activity against oral bacteria, and potentially reduce side effects, and therefore could emerge as an adjunct anti-biofilm treatment in the future. 

### 1.2. Periodontitis and Implant-Related Infections: Biofilm in Susceptible Hosts

Data from the 2018 NHANES estimated that 42% of dentate US adults have periodontitis [38], a chronic multifactorial infectious-inflammatory disease associated with polymicrobial biofilms and characterized by progressive destruction of the tooth-supporting structures [39]. Periodontitis is a significant public health problem, not only because of its high prevalence but also because it can lead to masticatory dysfunction, edentulism, and reduced patient quality of life [38]. In addition, periodontitis also leads to a low-grade systemic inflammatory burden that may enhance the risk or the severity of cardiovascular diseases, diabetes mellitus, and other systemic diseases [40,41].

Bacteria living in dental biofilms are the primary etiological factors of periodontal diseases [5,15]. However, as in the case of most human chronic infections, periodontitis onset and progression are not only dependent on the presence of specific microorganisms but also on (i) a reduced proportion of host-compatible species, (ii) a susceptible host, and (iii) an altered local environment (e.g., presence of inflammation and deep pockets). Researchers still have the challenge of determining the temporal sequence and the minimal level of change in each of these factors that would lead to disease onset [42]. 

Although complex, the composition of the periodontal biofilm in health and disease has been extensively studied over decades [27]. It has been well established that periodontitis is associated with an imbalance between pathogens and beneficial microorganisms [5,23,27]. Our knowledge of the effects of different treatments in modulating the subgingival microbiome has also increased substantially in recent years. Overall, the data of these studies suggest that periodontal clinical improvements are associated with the suppression of pathogens and recolonization of the biofilm by host-compatible species [27,43]. The introduction of the new target and open-ended diagnostic tests in the 2000s and, more recently, of metatranscriptomic techniques, have enabled a more comprehensive evaluation of the biofilm composition, metabolic activity, and functionality [44,45,46]. Further studies using these technologies may enhance knowledge in this field and allow the establishment of more effective preventive and treatment strategies for periodontal disease. Some studies using sequencing technologies have suggested potential new periodontal pathogens such as *Filifactor alocis* [44,47]. *F. alocis* is known for its ability to reduce neutrophil functions, which allows the organism to survive inside the cell after phagocytosis. This species is considered an emerging oral pathogen with potential significant roles in the etiology of periodontitis, Refs. [48,49] as clinically isolated strains present extracellular vesicle proteins related to various virulence factors related to biofilm formation and effects on host cells [50]. 

Importantly, the oral biofilm formation process is a very complex process, and it varies on different sites (teeth, gingival tissue/mucosal surfaces and dental implanted materials), driven by nutritional, spatial, or metabolic factors, leading to highly complex and specialized communities mainly organized in biofilms [17]. Mechanistically, site-specific factors have been correlated with biofilm formation and composition, such as glucose [51] and calcium ion [52] availability, that seem to lead to an increase in bacterial-derived extracellular polysaccharide production and enhance biofilm biomass and metabolic activity, respectively. In dental implants, the presence of titanium ions seems to favor the development of a more virulent biofilm, harboring more pathogenic species, in vitro [53] Although clinically relevant, readers must keep in mind that these data come from in vitro models, and they cannot be directly translated into the clinic reality. In fact, microcosm models have been extensively used, by our group [54,55] and others [56], to reproduce oral microbiome composition using human saliva or oral biofilm collected in vivo as microbial inoculum to test in vitro situations. In this regard, recent evidence has shown that although cold storage conditions do not play a critical role in inoculum microbial composition, the subsequent selective environment for microbial growth may reduce the variation among a sample’s source and create a reproducible microcosm model [57]. 

In dental implants, similarly to periodontally involved teeth, polymicrobial biofilm infection has been considered the main reason for peri-implant disease, triggering exacerbated inflammatory response, and resulting in a loss of supporting structures [9,37,38,39,58,59,60]. These conditions, known as peri-implant mucositis, characterized by inflammation of the mucosa around dental implants, and peri-implantitis, followed by subsequent progressive loss of supporting bone, have the biofilm as the main etiologic factor [60]. Once exposed to the oral environment, the implant is immediately coated by a protein layer from biologic fluids (i.e., saliva), which mediate and promote microbial adhesion and accumulation [61].

The complex dynamics of implant biofilm-assembly comprise several events that modulate microbial accumulation [14]. If the healthy state of the biofilm-implant system disrupted, a microbiological shift with significant overgrowth of pathogenic and putative species may occur, leading to the development of implant-related infections [62]. Our group has found important factors modulating biofilm growth and virulence on implant surface, such as surface properties [55], carbohydrate exposure [63], extracellular matrix [54], and even the cross-kingdom interaction between bacteria and fungus [59]. Although implant-related infections are prevalent diseases that share certain similarities with the pathophysiology of periodontitis, these conditions are associated with a wide range of modulating factors that are still unknown and require further investigation.

The treatment of peri-implantitis remains a clinical challenge. Although some studies have reported favorable post-treatment results, others failed to show disease resolution. In addition, the progression or recurrence of peri-implantitis and implant loss after treatment have also been described [64]. In terms of maintenance, the most common supportive therapy following surgical treatment of peri-implantitis includes supra- and submucosal biofilm removal using titanium or carbon fiber curettes, or ultrasonic devices. Still, only 42% of treated implants following this maintenance protocol presented absence of bleeding after 5 years of follow-up [65]. Thus, novel strategies have been proposed to address these limitations, including the use of probiotics. *Lactococcus lactis*, for example, can produce nisin, a common antimicrobial agent often used for food preservation. Recently, it has been shown that *L. latics* and nisin presence during biofilm formation, in vitro, shifted the composition, relative abundance, and diversity levels of these biofilms formed over titanium discs toward a healthy state, showing that this could be a future avenue for peri-implantitis treatment [66].

In addition to the therapeutic strategies, the development of antifouling biomaterials has shown to be a promising strategy to control microbial accumulation and inflammatory processes [67]. Since physical–chemical properties of biomaterial surfaces modulate microbial adhesion, and topographical patterns play a role in the dynamics of biofilm growth [67], surface modifications have been suggested to enhance biological responses [55,68]. Although it has not been translated to clinical practice, the current evidence from animal models suggests that drug-loaded materials may enhance bacterial killing [10]. However, most studies have shown drug release within hours or days, which may be an obstacle in the treatment of a chronic disease such as peri-implantitis. To overcome this problem, smart biomaterials with a drug releasing under on-demand activation (i.e., pH or temperature variation) coatings may provide a promising strategy to control microbial accumulation and the inflammatory process at the right moment and site [11].

### 1.3. Oropharyngeal Candidiasis: Microbial and Fungal Cross-Kingdom Interactions

Oropharyngeal candidiasis (OPC) is the most prevalent fungal infection in patients with weakened or immature immune systems, such as neonates [69], HIV+ children [70], and adults [71], and patients undergoing treatment for head and neck cancer therapy [72]. Candida bloodstream infection (candidemia) is a common severe systemic infection that mainly develops in patients under intensive medical care, in which the mortality rate can be over 27% [22]. 

Previous studies by our group have shown the influence of *C. albicans* infection on the composition of the oral mucosa-associated bacteria in the context of cytotoxic chemotherapy [20,21]. We demonstrated that *C. albicans* infection led to a profound taxonomic imbalance in the oral mucosa that contributed to increased biofilm virulence and oral mucosal invasion. Notably, mucosal injury and immunosuppression caused by chemotherapy significantly increased virulence and invasive infection in this model, with dysbiotic communities playing an accessory role.

Among dietary factors, frequent consumption of fermentable carbohydrates, such as sucrose, has been shown to strongly influence the ecology of the oral biofilm, leading to decreased species richness due to a significant increase in the abundance of streptococci [51]. Thus, more recently, we showed how a high sucrose diet could create a microbiome shift promoting the colonization of certain acidogenic and aciduric oral streptococci, which have been previously reported, by our group and others, to have a mutualistic relationship cross-kingdom interaction with *C. albicans* [73,74,75]. 

As more information emerges on the oral microbiota using advanced sequencing methodologies, it is imperative to examine how organisms modulate their capacity to colonize or trigger infection. Unfortunately, most mouse models of oral *C. albicans* infection have focused on interactions with single bacterial species, and the role of microbiome shifts that could lead to Candida pathogenicity has been disregarded. Thus, there is still a gap in understanding the relationship between Candida and the oral bacterial microbiome. We propose that certain oral commensal bacteria contribute to fungal pathogenesis while others have an antagonistic effect [3,20,21,73,74]. Although our research has helped to identify gaps in our understanding of the mechanisms involved in these synergistic and antagonistic interactions, future studies in this field are still necessary. 

Although it is well known that the microbiome exerts widespread influences on the control and development of immune responses, the cross-talk between the oral and gut microbiome through the systemic immune response is still largely unknown. Recently, researchers have shown that germ-free mice are highly susceptible to *C. albicans* oral infection, whereas mice housed in standard conditions are fully resistant, showing the importance of commensal microbiota in preventing fungal colonization. Strikingly, the co-colonization of oral Candida and gut segmented filamentous bacteria (SFB), a Gram-positive microbe that colonizes the distal ileum, was sufficient to restore immune protection against *C. albicans* in the oral cavity of germ-free mice. SFB co-colonization induced IL-17 expression in oral T-lymphocytes, paired with the increase in oral b-defensins and neutrophil-recruiting chemokines, reducing oral susceptibility to candidiasis. These findings provide new insights into the importance of beneficial microorganisms in preventing fungal infections [76].

## 2. Future Challenges

Oral microbial communities may live in a homeostatic condition with the host. However, this equilibrium can be lost under certain conditions, triggering biofilm-associated diseases. As pointed out in this Editorial, despite our current broad understanding of the role of oral biofilms in the onset and progression of several oral diseases, there is still room for improvement in this area. For example, the composition and structure of the biofilms and the detailed immune-inflammatory mechanisms/pathways on how oral microorganisms interact with the host to transit from health to disease is still under investigation. Worth noting that the increased resistance of fungi and bacterial species to antimicrobial drugs is a critical healthcare issue, making research in the field of oral biofilms highly relevant to public health. Moreover, the evaluation of microbial dynamics related to the oral diseases needs to be conducted under standardized methods, mainly under conditions that can closely mimic the oral environment, when in vivo human studies are not possible. 

Finally, to fill the gaps in knowledge, the oral biofilm composition, structure, functionality, and the keystone modulatory properties and virulent factors of colonizing species should be dissected with a scalpel. There is still a rich vein of research on this topic for years ahead. We are confident that this special issue of “*Microorganisms*” will be a step forward toward defining more efficient treatment strategies for controlling oral biofilm-associated diseases.
